# Analysis of the immediate effect of the high-frequency oral oscillation exercise on individuals with and without vocal symptoms

**DOI:** 10.1590/2317-1782/e20240056en

**Published:** 2025-02-07

**Authors:** Miriã Isabela dos Santos Dantas, Ana Cristina Côrtes Gama, Lorena Luiza Costa Rosa Nogueira, Renata Maria Moreira Moraes Furlan

**Affiliations:** 1 Programa de Pós-graduação em Ciências Fonoaudiológicas, Universidade Federal de Minas Gerais – UFMG - Belo Horizonte (MG), Brasil.; 2 Departamento de Fonoaudiologia, Faculdade de Medicina, Universidade Federal de Minas Gerais – UFMG - Belo Horizonte (MG), Brasil.

**Keywords:** Voice, Vocal Quality, Speech Acoustics, Voice Training, Speech, Language and Hearing Sciences

## Abstract

**Purpose:**

to verify the immediate effects of high-frequency oral oscillation using the Classic Shaker®, on acoustic, perceptual-auditory and vocal self-perception measures, in adults with and without vocal complaints.

**Methods:**

50 individuals were allocated into four groups - men with vocal symptoms, men without vocal symptoms, women with vocal symptoms and women without vocal symptoms. The participants completed the Vocal Symptoms Scale, the self-assessment of vocal discomfort, and were subjected to voice recording before and after performing the tested exercise, which consisted of blowing the Shaker® - Classic model - mouthpiece, while emitting the vowel U, for three minutes. The recordings were submitted to acoustic analysis and perceptual-auditory analysis. Paired T-test and Wilcoxon test were used, significance level of 5%.

**Results:**

after the exercise, there was a decrease in jitter in the groups of men with symptoms and in shimmer in men without symptoms. Women with symptoms showed an increase in fundamental frequency, harmonic-to-noise ratio, CPP, and CPPS values and a decrease in jitter; women without symptoms showed an increase in GNE. The perceptual-auditory evaluation did not indicate changes after carrying out the exercise. A reduction in vocal discomfort was observed in all groups after the exercise.

**Conclusion:**

The high-frequency oral oscillation exercise using the Shaker® was able to promote improvements in acoustic parameters and a reduction in self-reported vocal discomfort in the four groups evaluated.

## INTRODUCTION

Semi-Occluded Vocal Tract (SOVT) exercises are based upon a partial occlusion of the vocal tract during the vocalization process. Such exercises were originally used by singers and voice professionals, so to increase both performance and vocal quality^(1)^. Later on, these exercises were incorporated into Speech-Language Pathology clinical practice^(2)^. The SOVT exercises technique consists of reducing the vocal tract at the area closer to its end (the area that is closer to the lips). This technique determines the increase of the acoustic impedance^(2)^ related to the reactive component, more especially the positive reactance^(3)^, thus affecting the sound source. The SOVT exercises create changes in the way vocal folds vibrate, reducing muscular effort and assuring a more suitable vocal production^(2)^. Some examples of SOVT exercises are lips vibration techniques, prolonged “b”, glottic firmness, among others^(4)^.

The SOVT exercises have enormous acceptance among speech-language therapists^(5)^, because they reduce the phonatory effort^(6)^. Such strategies also promote vocal production balance by diminishing the glottal adduction force and by increasing the mucous membrane waving moves. This determines the balance between the larynx muscle contraction and the air flowing through it^(7)^. The SOVT exercises are also indicated for improving resonant balance, decreasing the noise of the acoustic larynx signal spectrum and increasing the number of amplified harmonics^(8).^

A new SOVT exercise by the name of Voiced Oral High-Frequency Oscillation (VOHFO)^(9)^ has been recently described. This exercise is performed using a device called Shaker®. This device was initially used in Physiotherapy for bronchial hygiene purposes^(10)^. Every Shaker® device model includes a nozzle at one end and a perforated cover at the opposite end, to allow for air exit. Inside the device there is a sphere made of high density stainless steel, which is supported by a circular cone^(10)^. Once the patient blows into the device, the stainless steel sphere vibrates^(9)^, and that changes the expired air flow. The mechanism works by resisting the patient’s blow. That causes the whole respiratory tract to shake, including the larynx^(10)^, which facilitates the mobilization of both bronchial and pulmonary secretions to the upper airway^(11)^.

As the exercise is executed, a partial obstruction occurs in the preceding area of the vocal tract. This process intensifies the interaction between source and filter, resulting in an increase of glottic and supraglottic pressure. As a consequence, there is a decrease in the amount of collision forces between vocal folds^(12)^. Furthermore, the oral oscillatory pressure takes place, caused by the second vibration source in the distal area of the vocal tract, that in the Voiced Oral High-Frequency Oscillation exercise happens to be the stainless steel sphere, thus creating a “massage” effect on the glottic area^(13-15)^.

Different models of the Shaker® device (Classic, New, and Plus) are commercially available, and a study indicates that differences among them can influence vocal parameters^(16)^. The usage of the New Shaker® for three minutes, associated with vocal emission, did create either positive or neutral sensations to the voice, larynx and breathing in elderly women^(9)^. Another study^(16)^, however, found that the aforementioned duration was beneficial for the exercise with the Plus Shaker® device for adults without vocal alterations, but not with the New Shaker®, as the use of the latter, after three minutes, increased symptoms of vocal fatigue. Antonetti et al.^(17)^ found that vocally healthy males better benefited from one minute of New Shaker® use than by a three-minute use. When it comes to the Classic model, Siqueira et al.^(18)^ found a decrease in vocal discomfort in women after a three-minute long exercise. Thus, the authors suggest three minutes to be the ideal exercise time with the Classic Shaker®, concerning the researched population^(18)^.

In spite of the existence of researches on the subject^(9,16-22)^, most of them were executed with the New Shaker®^(9,16,17,19-22)^. One study has been found to have used the Classic model^(18)^. Such study was carried out with females both with and without vocal complaints. The scarcity of research with the Classic model, the lack of information about its effects on vocal quality, and the absence of studies on male subjects amount to a knowledge gap. The present research aims to fulfill such void. Moreover, by incorporating acoustic and perceptual-auditory measures - as much as vocal self-perception, this research encompasses a multidimensional approach. Such an approach provides a broader view on the impact of the intervention upon the vocal function. This research expected results hold potential not only to increase scientific evidence on the issue, but also to contribute to good clinical practice. It will bring complementary information to that already existing in the literature about the Voiced Oral High-Frequency Oscillation exercise, in face of the Classic Shaker® device use.

The goal of the present study was to verify the immediate effects of Voiced Oral High-Frequency Oscillation by using the Classic Shaker® on acoustic and perceptual-auditory measures and on vocal self-perception, for adults with and without vocal symptoms.

## METHODS

Experimental research, with a pre-and-post intervention design, was conducted at the Functional Health Observatory on Speech-Language Pathology of the Federal University of Minas Gerais (UFMG) School of Medicine. The research was approved by the Research Ethics Committee of the UFMG (protocol number: 5.179.611, CAAE 53101021.0.00005149). All participants signed the Free Informed Consent Term. The research was registered at the REBEC platform under the protocol RBR-10s52xg3.

### Sample

The criteria used for inclusion were as follows: age between 18 and 60 years, and the presence or absence of vocal symptoms, depending on the participant group. The adopted criteria for exclusion were as follows: the presence of cardiovascular disease, either neurological or hearing diseases that could interfere in the phonation process, the presence of either lips or palate fissures, facial or costal arch fractures, superior air path obstruction at the experiment time, any serious kidney conditions, non-treated pneumothorax, smoking habits, having undergone prior speech-language therapy, or not having completed all steps of the study.

The study included 50 individuals, consisting of university employees and volunteers in general, with a mean age of 27.1 years (minimum of 18 and maximum of 56 years, and a standard deviation of 7.9 years), evenly distributed between 25 men and 25 women. This was a convenience sample. The sample size estimation was based on previous studies that have also investigated the effects of Voiced Oral high-frequency oscillation using the Shaker®^(9,19)^.

Participants were divided into four groups, accordingly to their results in the Voice Symptom Scale (VoiSS)^(23)^: males with vocal symptoms (VoiSS ≥ 16 points), males without vocal symptoms (VoiSS < 16 points), females with vocal symptoms (VoiSS ≥ 16 points), and females without vocal symptoms (VoiSS < 16 points). The VoiSS is a self-evaluating vocal protocol that provides information about the functionality, the emotional impact and the physical symptoms that vocal problems are likely to cause in an individual’s life^(23)^. The VoiSS consisted of 30 questions about vocal symptoms. There were five possible answers to each question: never, occasionally, some of the time, most of the time and always. Each question was scored from 0 (never) to 4 (always). Individuals achieving a score of 16 or higher were included into the “with vocal symptoms” group^(23)^.

The group of males with vocal symptoms consisted of 13 individuals. The mean age was 24.6 years; the standard deviation (SD) was 3.8, minimum of 19 and maximum of 34 years, and a mean VoiSS score of 26.46 (SD=10.33). The group of males with no vocal symptoms consisted of 12 individuals. The mean age was 25.1 years, with a SD of 6.0, a minimum of 10 and a maximum of 42 years and a mean VoiSS score of 9.08 (SD=3.23). Concerning female individuals, the group with vocal symptoms consisted of 13 individuals with a mean age of 29.5 years, a SD of 9.5, a minimum of 19 and a maximum of 53 years and a mean VoiSS score of 29.00 (SD=13.33). The group of females without symptoms consisted of 12 individuals with a mean age of 29 years, a SD of 10.1, a minimum of 21 and a maximum of 56 years, and a mean VoiSS score of 10.33 (SD=3.98).

### Procedures

First, participants filled in the Voice Symptom Scale (VoiSS) protocol^(23)^. Afterwards, they had their voices recorded. The recording equipment consisted of a laptop featuring an AMD Ryzen 5 3500U processor and a Dolby sound board; and a unidirectional Lesson® HD 74 microphone (cardioid). The microphone was duly connected to the computer and positioned laterally to the participants’ mouth at a five- centimeter distance. The sample rate was 44000 Hz and 16 bits per sample. Data collection was carried out inside an acoustically treated room. Participants sat at an upright posture, not only for the voice recording but for the exercise completion as well.

The participants’ voice records consisted of the sustained emission of the vowel /ɛ/, prolonged for the maximum phonation time, and the counting from 1 to 10. Five seconds were considered for the execution of the vowel /ɛ/. Both the initial and final excerpts were disregarded for acoustic voice analysis purposes. Such excerpts usually refer to a natural voice instability period. The analysis considered the middle section recorded, which lasted from three to four seconds.

After their vocal emissions (sustained vowel and counting), each participant classified his or her own vocal discomfort by the means of the Visual Numeric Scale (VNS), thus registering their self-perception at that given time. The VNS consists of a scale ranging from 0 to 10. At one end appears the number 0, meaning “no discomfort” and at the other end stands the number 10 meaning “maximum discomfort”. Participants were informed that “vocal discomfort” encompasses the discomfort, effort or tiredness they perceive in their voice at the present moment. Participants were told to mark on the scale the number that was better related to their self-perceived vocal discomfort at the time. Such marks were to be compared to the marks taken after the exercise execution.

Afterwards, participants executed an exercise by blowing into the Classic model Shaker® mouthpiece, with the emission of the vowel /u/. This exercise was executed at an usual frequency and with a continuous expiratory airflow which lasted for three minutes^(18)^, interrupted by pauses when needed. All participants performed the exercises while sitting on a chair and while having both their back and feet supported. Participants kept the Shaker® device on their lips with one hand, keeping a 90º angle between the device and the lip filter^(9).^ No previous training had taken place. However, all participants were provided with information on how to execute the exercises correctly.

After participants had practiced for three minutes, a new sequential recording of the /ɛ/ sustained vowel took place, followed by counting from 1 to 10, and a vocal discomfort self-perception register on the VNS.

The records obtained were subjected to acoustic analysis in the VoxMetria software, version 5.2. The analyzed parameters were mean fundamental frequency (f0) measured in Hz, noise (dB), glottal to noise excitation (GNE dB), jitter (%), shimmer (%), harmonic-to-noise ratio (dB), and the cepstral measures Cepstral Peak Prominence (CPP) and Cepstral Peak Prominence Smoothed (CPPS). The cepstral measures were obtained by the means of the Praat software, by following literature recommendations^(24)^. Thus, a comparative analysis was carried out for each acoustic parameter between the pre and post exercises periods.

The records were also subjected to the perceptual-auditory analysis of three speech-language therapists, all of them being voice experts with previous experience on voice perceptual-auditory analysis. The records were renamed as “voice A and voice B”, so to make for a blind analysis. The voices were sent to the speech-language therapists judges at a random order. This way the speech-language therapists analyzing the pairs of voices could not know if each voice being analyzed had been recorded either before or after the exercises. All analysis was independently made by the judges and, during the perceptual-auditory analysis, judges were oriented to listen to the voices as many times as they considered suitable. The headphones and computers used by the judges were not of the same brand or model.

The perceptual-auditory analysis was executed as a comparison task, so each judge was supposed to analyze, while listening to a pair of voices, if the second emission (Voice B) had “improved”, “worsened” or remained “unchanged” when related to the first voice (Voice A).

When there was a disagreement among the three judges over a particular pair of voices, an expert in voice speech-language therapist with over 20 years of experience served as a fourth appraiser. This last expert then evaluated the voices and determined the more concordant answer. Such analysis occurred for seven pairs of voices.

To determine the intra-judge’s agreement, 20% of the sample was randomly replicated. The Google Forms platform was used for gathering and organizing the answers provided by speech-language therapist judges.

### Data analysis

Statistical analysis was achieved by means of the statistical software MINITAB, version 17. First, a descriptive analysis of data with measures of central tendency and dispersion was executed. Calculations of absolute and relative frequencies were executed for the categorical variable (perceptual-auditory analysis). Later, the Anderson-Darling test was used to verify the sample normality. The paired t-test (parametric) or the Wilcoxon test (non-parametric) was used to compare the acoustic measurements of fundamental frequency (f0 Hz), noise (dB), glottal-to-noise excitation (GNE dB), jitter (%), shimmer (%), harmonic-to-noise ratio (dB), CPP, and CPPs, as well as vocal self-perception before and after exercise execution for each group. The Chi-square test was used to compare the four groups for the categorical variable perceptual-auditory evaluation. For this analysis, the “worsened” category was removed due to its low frequency at the notes (four for each task - sustained vowel and counting). A confidence level of 95% was used for all analysis. The power of the test for the variables that did not present statistically significant and the effect size for variables with p<0.05 were calculated by the G-Power software. The effect size was considered insignificant when below 0.19; small when between 0.10 and 0.49; medium for values between 0.50 and 0.79; and large when over 0.80^(25)^.

The intra-evaluator concordance on the perceptual-auditory analysis was evaluated by the means of the Gwet's AC1 statistics in the R version 3.3.1. software. The concordance degree was analyzed by considering: for values under zero - absent concordance; for values from 0 to 0.20 - small concordance; for values from 0.21 to 0.40 - weak concordance; for values from 0.41 to 0.60 - moderate concordance; for values from 0.61 to 0.80 - good concordance; and for values from 0.81 to 1.00 - almost perfect concordance^(26)^.

## RESULTS

Table 1 presents the acoustic measures and the vocal discomfort self-perception for the males with vocal symptoms’ group, before and after the exercise. It was verified a significant statistical difference for jitter (p=0.048) and vocal discomfort self-perception (p=0.009), with decreasing values for both variables after the exercise.

**Table 1:** Acoustic measures and vocal discomfort self-perception pre and post exercise on males with vocal symptoms (n=13)

**Variable**	**Pre-exercise**								**Post-exercise**							**p-value**	**1-β**	**Effect size**
	**Mean**	**SD**	**Median**	**1º** **quartile**		**3º quartile**	**Minimum**	**Maximum**	**Mean**	**SD**	**Median**	**1º** **quartile**	**3º quartile**	**Minimum**	**Maximum**			
Fundamental Frequency (Hz)	108.62	16.57	113.22	99.77	118.20		70.72	130.08	117.75	12.43	116.29	110.07	126.09	96.64	134.68	0.232^(A)^	0.827	-
Noise (dB)	1.68	0.79	1.61	1.17	1.79		0.53	3.54	1.38	0.65	1.34	1.00	1.84	0.45	2.52	0.106^(A)^	0.415	-
GNE (dB)	0.73	0.21	0.67	0.66	0.78		0.44	1.26	0.72	0.16	0.73	0.61	0.81	0.45	0.95	0.667^(A)^	0.672	-
HNR (dB)	7.89	4.83	8.52	6.21	10.82		1.04	16.31	8.54	5.66	9.91	2.45	11.27	0.52	17.6	0.711^(A)^	0.736	-
Jitter (%)	5.52	10.13	1.92	1.26	3.87		0.26	38.15	4.08	7.28	2.26	0.53	3.67	0.25	27.72	0.048*^(A)^	-	0.159
Shimmer (%)	33.0	26.19	23.75	19.41	36.49		12.64	114.10	20.75	9.15	23.25	11.37	24.55	6.73	36.23	0.507^(B)^	0.889	-
CPP vowel	23.18	3.38	23.56	21.35	24.97		17.35	28.13	24.83	3.11	24.94	23.08	27.52	19.56	29.49	0.055^(A)^	0.409	-
CPP count	19.64	1.20	19.41	19.15	20.55		17.41	21.71	19.78	1.16	19.99	18.79	20.49	17.90	21.82	0.734^(A)^	0.756	-
CPPS vowel	12.12	2.55	12.74	11.22	14.09		7.26	15.94	13.45	2.56	12.95	11.75	15.83	8.91	17.47	0.061^(^A^)^	0.447	-
CPPS count	10.49	1.41	10.69	10.18	11.27		6.6	12.21	10.39	1.29	10.42	9.66	11.52	8.15	11.94	0.713^(A)^	0.723	-
Vocal discomfort self-perception	2.38	2.14	2.0	0	4.0		0	6.0	0.61	0.77	0	0	1.0	0	2.0	0.009*(B^)^	-	0.943

Paired T Test^(A)^;

Wilcoxon Test^(B)^

* Significant p value (p≤ 0.05)

**Caption:** GNE=*Glottal to noise excitation*; HNR=harmonic-to-noise ratio; CPP=*Cepstral Peak Prominence;* CPPS=*Cepstral Peak Prominence-Smoothed*; 1-β=Power of the test

Table 2 presents the acoustic measures and the vocal discomfort self-perception for the males without symptoms’ group, before and after the exercise. It was verified a significant statistical difference for shimmer (p=0.042) and vocal discomfort self-perception (p=0.022), with decreasing values for both variables after the exercise.

**Table 2:** Acoustic measures and vocal discomfort self-perception pre and post exercise on males without vocal symptoms (n=12)

**Variable**	**Pre-exercise**							**Post-exercise**							**p-value**	**1-β**	**Effect size**
	**Mean**	**SD**	**Median**	**1º quartile**	**3º quartile**	**Minimum**	**Maximum**	**Mean**	**SD**	**Median**	**1º quartile**	**3º quartile**	**Minimum**	**Maximum**			
Fundamental Frequency (Hz)	104.97	39.23	116.44	103.42	122.40	77.40	162.11	102.79	42.17	108.2	97.92	123.71	72.01	177.12	0.820^(A)^	0.823	-
Noise (dB)	1.61	0.77	1.88	0.69	2.22	0.55	2.49	1.51	0.90	1.21	‘0.67	2.31	0.48	2.92	0.666^(B)^	0.690	-
GNE (dB)	0.64	0.17	0.59	0.50	0.72	0.46	0.92	0.69	0.22	0.76	0.50	0.89	0.35	0.94	0.388^(B)^	0.532	-
HNR (dB)	12.13	8.11	10.97	8.43	18.17	3.70	26.49	12.60	7.09	11.78	10.49	16.50	0.63	23.56	0.811^(A)^	0.815	-
Jitter (%)	3.19	3.80	1.33	0.70	4.3	0.34	9.84	3.02	4.16	1.00	0.59	3.43	0.2	14.38	0.290^(B)^	0.295	-
Shimmer (%)	24.58	13.79	20.98	15.71	35.97	3.22	47.27	17.91	10.33	16.33	11.32	20.68	5.05	39.53	0.042*^(A)^	-	0.537
CPP vowel	24.31	4.47	24.04	21.35	27.25	17.47	31.43	25.37	4.29	25.84	21.23	28.83	18.65	30.83	0.148^(A)^	0.271	-
CPP count	20.0	1.60	20.36	19.01	21.21	17.10	21.92	19.86	1.27	20.04	19.36	20.61	17.55	21.58	0.695^(A)^	0.710	-
CPPS vowel	13.11	3.59	12.68	10.60	15.66	7.01	18.81	14.08	3.57	14.43	10.53	17.40	8.55	18.59	0.078^(^A^)^	0.191	-
CPPS count	10.29	1.32	10.40	10.11	11.19	6.97	11.81	10.25	1.31	10.51	9.70	10.91	6.76	11.70	0.814^(B)^	0.815	-
Vocal discomfort self-perception	1.17	1.19	1.0	0	2.0	0	3.0	0.25	0.45	0	0	0.25	0	1	0.022*(B^)^	-	0.884

Paired T Test^(A)^;

Wilcoxon Test^(B)^

* Significant p value (p≤ 0.05)

**Caption:** GNE=*Glottal to noise excitation*; HNR=harmonic-to-noise ratio; CPP=*Cepstral Peak Prominence;* CPPS=*Cepstral Peak Prominence-Smoothed*; 1-β=Power of the test

Table 3 presents the acoustic measures and the vocal discomfort self-perception for the women with vocal symptoms’ group, before and after the exercise. After the exercise there was an increase of the fundamental frequency (p=0.010), the harmonic-to-noise ratio (p=0.011), the CPP (p=0.002), and the sustained vowel CPPs (p=0.014); and a jitter (p=0.014) and vocal discomfort (p=0.002) decrease.

**Table 3:** Acoustic measures and vocal discomfort self-perception pre and post exercise on females with vocal symptoms (n=13)

**Variable**	**Pre-exercise**							**Post-exercise**							**p-value**	**1-β**	**Effect size**
	**Mean**	**SD**	**Median**	**1º** **quartile**	**3º quartile**	**Minimum**	**Maximum**	**Mean**	**SD**	**Median**	**1º** **quartile**	**3º quartile**	**Minimum**	**Maximum**			
Fundamental Frequency (Hz)	196.51	27.16	205.94	179.58	212.67	130.37	239.88	207.97	23.93	204.72	200.14	219.72	158.24	253.04	0.010*^(A)^	-	0.446
Noise (dB)	1.28	0.65	1.06	0.82	1.78	0.39	2.59	1.09	0.57	1.09	0.56	1.30	0.41	2.08	0.080^(A)^	0.242	-
GNE (dB)	0.75	0.16	0.80	0.63	0.86	0.43	0.96	0.79	0.14	0.79	0.74	0.92	0.55	0.96	0.089^(A)^	0.216	-
HNR (dB)	9.67	7.54	7.47	5.21	13.45	0.66	28.19	12.67	7.01	11.78	6.37	15.02	5.54	27.93	0.011*^(A)^	-	0.411
Jitter (%)	3.59	4.24	2.11	0.73	5.12	0.19	14.12	1.80	2.73	1.51	0.19	2.06	0.08	10.36	0.014*^(B)^	-	0.480
Shimmer (%)	16.60	12.66	12.43	9.05	16.1	2.1	49.05	10.59	6.93	8.82	5.78	13.00	2.56	24.86	0.184^(B)^	0.700	-
CPP vowel	23.56	2.29	23.26	21.85	24.75	21.01	28.53	24.36	2.35	23.47	22.90	26.49	20.89	28.22	0.002*^(B)^	-	0.345
CPP count	19.19	1.45	19.45	18.23	19.88	16.71	21.94	19.40	0.85	19.41	19.13	19.95	17.79	20.79	0.616^(A)^	0.674	-
CPPS vowel	12.81	2.47	12.09	11.47	14.4	9.47	17.84	13.63	2.39	12.92	12.06	16.22	10.27	17.29	0.014*^(B)^	-	0.337
CPPS count	9.77	0.92	9.90	9.52	10.24	7.32	11.04	10.05	0.80	9.91	9.48	10.48	8.99	11.46	0.198^(^A^)^	0.443	-
Vocal discomfort self-perception	4.15	2.54	5.0	2.0	6.0	1.0	8.0	1.61	2.06	0	0	3.0	0	5.0	0.002*(B^)^	-	1.087

Paired T Test^(A)^;

Wilcoxon Test^(B)^

* Significant p value (p≤ 0.05)

**Caption:** GNE=*Glottal to noise excitation*; HNR=harmonic-to-noise ratio; CPP=*Cepstral Peak Prominence;* CPPS=*Cepstral Peak Prominence-Smoothed*; 1-β=Power of the test

Table 4 presents the acoustic measures and the vocal discomfort self-perception for the women with no vocal symptoms’ group, before and after the practice of the exercise. There was a significant difference for the variables GNE (p=0.013) and vocal discomfort (p=0.009). It was verified an increase of the GNE and a decrease of the vocal discomfort.

**Table 4:** Acoustic measures and vocal discomfort self-perception pre and post exercise on females without vocal symptoms (n=12)

**Variable**	**Pre-exercise**							**Post-exercise**								**p-value**	**1-β**	**Effect size**
	**Mean**	**SD**	**Median**	**1º quartile**	**3º quartile**	**Minimum**	**Maximum**	**Mean**	**SD**	**Median**	**1º quartile**		**3º quartile**	**Minimum**	**Maximum**			
Fundamental Frequency (Hz)	237.24	89.10	212.44	189.59	229.32	184.08	273.47	214.47	22.18	209.72	201.67	232.54		181.09	253.19	0.733^(A)^	0.831	-
Noise (dB)	1.30	0.71	1.30	0.75	1.65	0.42	2.87	1.02	0.40	1.03	0.67	1.25		0.49	1.67	0.085^(A)^	0.400	-
GNE (dB)	0.72	0.16	0.74	0.66	0.87	0.36	0.91	0.81	0.10	0.81	0.75	0.90		0.65	0.94	0.013*^(A)^	-	0.643
HNR (dB)	13.57	7.38	13.09	10.61	17.29	2.22	26.52	14.87	7.48	13.52	10.08	18.02		4.72	31.50	0.147^(A)^	0.212	-
Jitter (%)	1.94	2.54	1.02	0.39	1.65	0.13	7.40	1.91	2.46	0.70	0.54	1.98		0.14	7.13	0.845^(B)^	0.845	-
Shimmer (%)	10.76	4.81	11.10	7.45	14.37	2.54	16.97	12.47	6.50	12.15	9.28	15.68		2.35	25.68	0.335^(A)^	0.536	-
CPP vowel	23.04	2.12	23.55	21.09	24.75	19.74	25.59	23.97	1.97	23.80	22.60	25.36		20.60	27.68	0.060^(A)^	0.332	-
CPP count	18.86	2.12	18.83	17.36	20.63	15.90	21.54	18.49	1.75	18.46	17.29	20.11		15.83	20.65	0.181^(A)^	0.263	-
CPPS vowel	12.71	2.07	12.82	10.79	14.26	10.04	15.62	13.48	2.06	13.73	11.97	14.58		10.13	17.47	0.066^(^A^)^	0.260	-
CPPS count	9.31	1.67	10.09	8.33	10.25	6.33	11.08	9.18	1.80	9.98	7.86	10.70		6.06	10.87	0.367^(B)^	0.382	-
Vocal discomfort self-perception	2.50	1.57	2.50	2.0	3.0	0	5.0	0.83	1.40	0.5	0	1.0		0	5.0	0.009*(B^)^	-	1.119

Paired T Test^(A)^;

Wilcoxon Test^(B)^

* Significant p value (p≤ 0.05)

**Caption:** GNE=*Glottal to noise excitation*; HNR=harmonic-to-noise ratio; CPP=*Cepstral Peak Prominence;* CPPS=*Cepstral Peak Prominence-Smoothed*; 1-β=Power of the test

Table 5 presents the result of the perceptual-auditory analysis for the four studied groups, considering a comparison between the voices at the post and pre-exercise moments. A 100% intra-evaluator concordance was verified for the three judges. This way, the answers given by the three appraisers were considered for the voice perceptual-auditory analysis, so the mode value from the answers given by the speech-language therapists was used. For the most part of participants, no vocal alterations were verified at the perceptual-auditory evaluation, and no difference was verified between groups (p=0.379) for the sustained vowel. There was, however, a difference between groups for the connected speech (p=0.003). The group of women with vocal symptoms was the group presenting the lesser number of vocal alterations after the Voiced Oral High-Frequency oscillation exercise.

**Table 5:** Pre-exercise and post-exercise voice comparison results, by the means of auditory perception analysis

**Result**	**Sustained vowel**		**Count**	
	**N**	**%**	**N**	**%**
Males with vocal symptoms (n=13)				
Improved	9	69.23	6	46.15
Unchanged	4	30.77	7	53.85
Worsened	0	0	0	0
Males without vocal symptoms (n=12)				
Improved	5	41.67	3	25
Unchanged	6	50	9	75
Worsened	1	8.33	0	0
Females with vocal symptoms (n=13)				
Improved	5	38.46	0	0
Unchanged	7	53.85	13	100
Worsened	1	7.69	0	0
Females without vocal symptoms (n=12)				
Improved	3	25	0	0
Unchanged	7	58.33	8	66.67
Worsened	2	16.67	4	33.33

**Caption:** N=absolute frequency; %=relative frequency

## DISCUSSION

The present research indicates that the VOHFO exercise executed with the Classic Shaker*®* promoted positive vocal changes, verified at the acoustic and self-perception analysis in adults. Such changes varied according to gender and the presence of vocal symptoms. Women with vocal symptoms presented positive results on a higher number of acoustic measures after the exercise. All groups’ self-perception statements indicated less vocal discomfort, suggesting that the VOHFO exercise performed with Classic Shaker*®* is a safe exercise.

Concerning the males with vocal symptoms, there was a *jitter* reduction and a decrease on vocal discomfort after the Voiced Oral High-Frequency Oscillation exercise. Considering that *jitter* is a short time fundamental frequency variation and that it determines the phonatory systems’ constancy, lower values relating to this acoustic measure after the exercise do suggest a bigger stability and a smaller fundamental frequency perturbation^(27)^. Saters et al.^(19)^, using the New Shaker®, pointed to the decrease of both vocal and larynx symptoms in males with vocal symptoms. No difference in acoustic parameters was verified after a three-minute exercise execution, though^(19)^. Marotti et al.^(28)^, also by using the New Shaker®, found no significant perceptual-auditory changes in the mentioned population, either for vowel or number counting analysis. They verified, however, self-perceived immediate positive sensations related to voice, larynx, breathing and articulation. This way, the studies agree upon the positive effects of VOHFO exercise as a SOVT exercise in reducing vocal discomfort. Such results suggest this exercise favors balance between larynx muscular contractions and the exhaled air flow^(7)^, thus relieving vocal symptoms discomfort.

In the males with no vocal symptoms’ group there was a decrease on *shimmer*, in addition to a decrease in the self-reported vocal discomfort. The *shimmer* classifies the short-time variability in the amplitude of the sound wave^(27)^, thus being a voice intensity disturbance measure. The practice of the researched exercise is characterized by the partial occlusion of the vocal tract anterior region. It is licit to imply that the Shaker® increases the source-filter interaction, provokes the decrease of glottic pressure, and, consequently, favors the amplitude periodicity of vocal fold vibration; in addition to also improving vocal comfort^(9,18,19)^. A study^(21)^ on the New Shaker® found a decrease of the *shimmer* on women without larynx symptoms after a five-minute VOHFO exercise. It can indicate that VOHFO exercise promotes bigger stability of vocal fold vibration in individuals with no vocal symptoms. The findings of the present research concerning the improvement of vocal discomfort in males with no vocal symptoms agree with the findings of another study^(20)^ which, by using the New Shaker®, verified a decrease on larynx symptoms after the execution of this same exercise.

The group of women with vocal symptoms was the one that presented positive acoustic changes on a bigger number of variables. It was verified an increase of fundamental frequency, harmonic-to-noise ratio, vowel cepstral measures and a decrease on both *jitter* and vocal discomfort. A study on the Classic Shaker® with women with and without vocal symptoms found only *jitter* decrease and an improvement on vocal discomfort ^(18)^. Another study, that employed the New Shaker®, verified improvement on vocal and larynx symptoms^(19)^ on normophonic and dysphonic women. An increase in fundamental frequency was observed in individuals without vocal complaints, in the study by Saters et al.^(19)^, using the New Shaker®, as a response to VOHFO exercises. This finding was also reported in studies involving other semi-occluded vocal tract exercises^(8)^.

It was verified an increase of CPP and CPPs values evaluated by the vowel, concerning the woman with vocal symptoms’ group. CPP is a cepstral measure, procedure of the extraction of fundamental frequency from a sound wave spectrum^(29)^. The Cepstrum shows, in the form of a chart, how spectral harmonics - particularly the fundamental vocal frequency - are individualized and highlighted from background noise^(29)^. The more regular the harmonic peaks and the bigger the periodicity and the general voice signal energy are, the bigger the cepstral peak^(29)^ will be. This way, the increase on both CPP and CPPs verified on women with vocal symptoms after the execution of the exercise indicates vocal quality improvement, resulting from the increasing of the harmonic structure of the voice. Studies point that cepstral measures have been displaying potential, especially for broad deviation range voice evaluation^(30,31)^, which justifies why only the women with vocal symptoms presented an increase on such measures. It is worthy to highlight that sustained vowel CPP and CPPs values for the males with vocal symptoms were close to the cutoff point for statistical significance. It is possible that a different exercise execution time for men with vocal symptoms would bring positive results for the cepstral measures. Antonetti et al.^(17)^ verified that both males and females without vocal alterations had an increase of CPPs, starting one minute after performing the VOHFO exercise with the New Shaker®, with the increase maintained after three minutes of the exercise. No studies were found, however, that evaluated cepstral measures in males with symptoms or even on dysphonic males both before or after the exercise.

A GNE increase and a vocal discomfort decrease were verified for the women without vocal symptoms’ group. While the literature agrees upon the fact that the VOHFO exercises for normophonic women, with the New Shaker® device, promote an improvement on vocal symptoms^(9,19)^ and promote vocal comfort^(18)^, the GNE variation was not a finding verified at any other study about the Shaker®. The GNE acoustic measure is directly related to the presence of roughness and breathiness^(32)^. Lower values of GNE can indicate ineffective glottic closing, with the presence of voice noise and a possible loss of intensity^(32)^. In this sense, the increase of GNE is a positive result for this group.

During the auditory-perception analysis no vocal alteration was verified after the execution of the exercise for most participants. In spite of the males with vocal symptoms’ group presenting a higher number of individuals with vocal improvement at the judge’s judgment, there was no significant difference among the groups. The present research’s result agrees with the literature. A study^(9)^ with elderly women pointed to a larger amount of individuals who kept their vocal quality after executing the exercise for a three-minute time. Other studies on adults with no vocal complaints^(16)^ and dysphonic adults^(22)^ have also verified an absence of changes on the voice auditory-perception analysis after an execution of the exercise for three^(16,22)^, five^(22)^ and seven minutes^(22)^. The three studies^(9,16,22)^ were executed with the New Shaker®.

It is highlighted that the ideal exercise execution time may not be the same for both genders, and this may have influenced the findings. The current research was guided by the Siqueira and collaborators’ study^(18)^, which evaluated the ideal time for the exercise execution with the Classic Shaker® for females only. Another study with the New Shaker® verified a significant decrease on the acoustic vocal noise parameter for healthy males after seven minutes of VOHFO exercise execution^(21)^. Regarding females, from the third minute on it was verified meaningful differences in larynx and pharynx symptoms^(21)^. More studies with the Classic model are needed to evaluate the effect of the VOHFO exercise on the masculine population for a longer execution time, aiming at a better understanding of the ideal vocal exercise dose for that population.

The effect size ranged from insignificant to large, with vocal discomfort self-perception showing a large effect size in all four tested groups^(25)^. Literature does not present which effect size can be considered relevant for the researched variables. In this sense, it is important that the Speech-Language Pathology starts focusing in the effect size analysis matter, aiming for a bigger understanding of the effect size that is clinically relevant to the area.

Literature has shown that semi-occluded vocal tract exercises can be used for both voice quality improvement training and vocal warm-up^(9,19)^. Therefore, studies already published, as well as the present research results, suggest that this is a safe exercise, under the clinical aspects evaluated, as the exercise did not worsen either vocal discomfort or the controlled parameters. It is suggested that future research evaluate the VOHFO exercise effect on voice professionals as well.

This study faced the following limitations: convenience recruitment and a sample size that, on being stratified, resulted in few individuals per group. This caused the power of the test to become, for some variables, below the recommended level. Another limitation was the wide age range of the sample, given that vocal changes can occur during the climacteric period^(33)^. One more important limitation concerns the group division being based upon a self-evaluation scale. This way, it is possible that, among the individuals with no vocal symptoms, there were dysphonic individuals. The larynx image was not a variable on this research. Therefore, future research with larynx exams of participants can bring important contributions concerning the voiced oral high-frequency oscillation exercise effects on the glottic functions. It is also suggested the research of measures that can offer information on the source-filter rate, such as the alpha ratio, L1-L0, for example. Despite such limitations, the present study brings its contribution to the Speech-Language Pathology clinical practice, given the methodological rigorousness applied on its development and the scarcity of studies using the Classic Shaker® that includes cepstral measures analysis. It is suggested, in addition, research into larger samples, so to allow the verification of the real effect of VOHFO exercises on individuals with different clinical conditions and that present a larger external validity.

It is recommended that further research be conducted to analyze the effect of the VOHFO exercises on dysphonic participants, as well as the effects of the device in the long term. The association of clinical larynx exams to such studies can also enrich the findings, enabling for a multi-dimensional analysis of the voice. Additionally, it is important to conduct new studies comparing different exercise time durations for both males and females, as well as for different clinical conditions and age groups.

## CONCLUSION

The voiced oral high-frequency oscillation exercise using the Classic Shaker® device and performed for three minutes promoted immediate positive acoustic changes for all analyzed groups. Perceptual-auditory changes were not verified. All groups reported a reduction in vocal discomfort.
