# Supplementary material for: Analysis of the immediate effect of the high-frequency oral oscillation exercise on individuals with and without vocal symptoms
Source: Codas. 2025 Feb 7;37(2):e20240056. doi: 10.1590/2317-1782/e20240056en (PMC11813183; doi:10.1590/2317-1782/e20240056en)
Supplement: Translation

# Translation: Análise do efeito imediato do exercício de oscilação oral de alta frequência sonorizada em indivíduos com e sem sintomas vocais

**DOI:** 10.1590/2317-1782/e20240056pt

**Authors:** Miriã Isabela dos Santos Dantas, Ana Cristina Côrtes Gama, Lorena Luiza Costa Rosa Nogueira, Renata Maria Moreira Moraes Furlan

**Affiliations:** 1 Programa de Pós-graduação em Ciências Fonoaudiológicas, Universidade Federal de Minas Gerais – UFMG - Belo Horizonte (MG), Brasil.; 2 Departamento de Fonoaudiologia, Faculdade de Medicina, Universidade Federal de Minas Gerais – UFMG - Belo Horizonte (MG), Brasil.

**Keywords:** Voz, Qualidade da Voz, Acústica da Fala, Treinamento da Voz, Fonoaudiologia

## Abstract

**Objetivo:**

verificar os efeitos imediatos da oscilação oral de alta frequência sonorizada utilizando o dispositivo Shaker® Classic, nas medidas acústicas, perceptivo-auditivas e autopercepção vocal, em adultos com e sem sintomas vocais.

**Método:**

participaram 50 indivíduos, distribuídos em quatro grupos - homens com sintomas vocais, homens sem sintomas vocais, mulheres com sintomas vocais e mulheres sem sintomas vocais. Os participantes preencheram a Escala de Sintomas Vocais, uma escala visual numérica quanto ao nível de desconforto vocal e foram submetidos à gravação da voz antes e após a execução do exercício testado, o qual consistiu em soprar o bocal do Shaker® - modelo Classic, ao mesmo tempo em que emitiam a vogal U, durante três minutos. As gravações foram submetidas à análise acústica e análise perceptivo-auditiva. Foram utilizados os testes T-pareado e de Wilcoxon, com nível de significância de 5%.

**Resultados:**

após o exercício houve diminuição do jitter no grupo de homens com sintomas vocais e do shimmer no de homens sem sintomas vocais. Mulheres com sintomas vocais apresentaram aumento da frequência fundamental, da proporção harmônico-ruído, dos valores de CPP e CPPS e diminuição do jitter; mulheres sem sintomas vocais apresentaram aumento de GNE. A avaliação perceptivo-auditiva não indicou mudanças após a realização do exercício. Observou-se redução do desconforto vocal em todos os grupos, após o exercício.

**Conclusão:**

o exercício de oscilação oral de alta frequência sonorizada, utilizando o Shaker®, foi capaz de promover melhora em parâmetros acústicos e diminuição de desconforto vocal autorreferido nos quatro grupos avaliados.

## INTRODUÇÃO

Os exercícios de trato vocal semiocluído (ETVSO) são baseados na oclusão parcial do trato vocal durante a vocalização e foram originalmente utilizados por cantores e profissionais da voz como forma de aumentar o desempenho e a qualidade vocal^(1)^. Posteriormente, foram incorporados à terapia fonoaudiológica^(2)^. A técnica de execução dos ETVSO consiste em reduzir a área do trato vocal em sua extremidade (mais próxima aos lábios), o que determina o aumento da impedância acústica^(2)^ em relação ao componente reativo, mais especificamente a reatância positiva^(3)^, o que afeta a fonte sonora. Assim, os ETVSO promovem mudanças nos modos de vibração das pregas vocais, reduzindo o esforço muscular e garantindo uma produção vocal mais adequada^(2)^. São exemplos de ETVSO as técnicas de vibração de lábios, “b” prolongado, firmeza glótica, dentre outros^(4)^.

Os ETVSO têm enorme aceitação entre os fonoaudiólogos^(5)^, pois reduzem o esforço fonatório^(6)^. Tais estratégias também promovem o equilíbrio da produção vocal, com diminuição da força de adução glótica e aumento do movimento ondulatório da mucosa, determinando o equilíbrio entre as contrações musculares da laringe e do fluxo aéreo que passa pela mesma^(7)^. Há, ainda, a indicação de ETVSO para melhorar o equilíbrio ressonantal, diminuindo o ruído do espectro do sinal acústico laríngeo e aumentando o número de harmônicos amplificados^(8)^.

Recentemente, foi descrito um novo exercício de trato vocal semiocluído, denominado oscilação oral de alta frequência sonorizada (OOAFS)^(9)^, realizado por meio de um dispositivo denominado Shaker®, inicialmente utilizado pela Fisioterapia para higiene brônquica^(10)^. O dispositivo Shaker®, independente do modelo, tem um bocal em uma extremidade e uma tampa com orifícios para a saída de ar na outra extremidade. Em seu interior, há uma esfera composta por aço inoxidável de alta densidade, suportada por um cone circular^(10)^. Uma vez que o paciente sopra o dispositivo, a esfera de aço inoxidável vibra^(9)^, o que altera o fluxo de ar expirado. O mecanismo funciona por resistência ao sopro, que causa vibração de todo o trato respiratório, incluindo a laringe^(10)^, o que facilita, também, a mobilização das secreções brônquicas e pulmonares para regiões da via aérea superior (VAS)^(11)^.

Durante sua realização, ocorre uma obstrução parcial na região anterior do trato vocal. Esse processo intensifica a interação entre a fonte e o filtro, resultando no aumento da pressão glótica e supraglótica. Como consequência, há uma redução da força de colisão entre as pregas vocais^(12)^. Além disso, a pressão oral oscilatória, causada pela segunda fonte de vibração na parte distal do trato vocal, que no caso do exercício de oscilação oral de alta frequência sonorizada é a esfera de aço inoxidável, origina um efeito de “massagem” na região glótica^(13-15)^.

Encontram-se disponíveis comercialmente diferentes modelos do dispositivo Shaker® (*Classic, New e Plus*) e um estudo aponta que existem diferenças entre eles capazes de influenciar parâmetros vocais^(16)^. O uso do *New* Shaker® por três minutos associado à emissão vocal provocou sensações positivas ou neutras para voz, laringe e respiração em mulheres idosas^(9)^. Outro estudo^(16)^, no entanto, verificou que o tempo em questão foi benéfico para o exercício com uso do dispositivo Shaker® *Plus* para adultos sem alterações vocais, mas não com o *New* Shaker®, sendo que o uso deste último, após três minutos, aumentou sintomas de fadiga vocal. Antonetti et al.^(17)^ encontraram que homens vocalmente saudáveis se beneficiaram mais do uso do *New* Shaker® por um minuto de execução do que por três minutos. Já com relação ao modelo *Classic*, Siqueira et al.^(18)^ verificaram diminuição do desconforto vocal em mulheres, após três minutos de realização do exercício. Dessa forma, os autores sugerem que três minutos é o tempo ideal de realização do exercício, para a população pesquisada, utilizando o Shaker® *Classic* ^(18)^.

Apesar de existirem pesquisas sobre o tema^(9,16-22)^, a maioria foi realizada utilizando o *New* Shaker®^(9,16,17,19-22)^. Apenas um estudo utilizando o modelo *Classic* foi encontrado^(18)^, o qual foi realizado com mulheres com e sem queixa vocal. Dessa forma, a escassez de pesquisas com o modelo *Classic*, a ausência de informações sobre os seus efeitos na qualidade vocal e a ausência de estudos em indivíduos do sexo masculino para este modelo criam uma lacuna de conhecimento que esta pesquisa busca preencher. Além disso, ao incorporar medidas acústicas, perceptivo-auditivas e a autopercepção vocal, a pesquisa engloba uma abordagem multidimensional, proporcionando uma visão mais abrangente dos impactos dessa intervenção na função vocal. Os resultados esperados têm o potencial não apenas de aumentar a evidência científica sobre o tema, mas também de contribuir para a prática clínica, com informações complementares às que já existem na literatura sobre o exercício de OOAFS, frente ao uso do dispositivo Shaker®, modelo *Classic*.

O objetivo deste estudo foi verificar os efeitos imediatos da oscilação oral de alta frequência sonorizada utilizando o dispositivo Shaker*®* *Classic,* nas medidas acústicas, perceptivo-auditivas e autopercepção vocal, em adultos com e sem sintomas vocais.

## MÉTODO

Foi realizada uma pesquisa, de delineamento experimental, com estudo de intervenção do tipo antes e após, no Observatório de Saúde Funcional em Fonoaudiologia da Faculdade de Medicina da UFMG. A pesquisa foi aprovada pelo Comitê de Ética em Pesquisa da Universidade Federal de Minas Gerais (número do parecer: 5.179.611, CAAE 53101021.0.00005149). Todos os participantes assinaram o Termo de Consentimento Livre e Esclarecido (TCLE). A pesquisa foi registrada na plataforma REBEC sob o protocolo RBR-10s52xg3.

### Amostra

Constituíram critérios de inclusão: ter idade entre 18 e 60 anos e apresentar ou não sintomas vocais, a depender do grupo de cada participante. Os critérios de exclusão foram: presença de doença de origem cardiovascular, doença neurológica ou auditiva que pudessem interferir no processo de fonação, presença de fissura de lábios e/ou palato, de fraturas faciais ou de arcos costais, de obstrução de VAS no momento da coleta, de alterações renais graves, de pneumotórax não tratado, ser tabagista, ter realizado fonoterapia prévia e não completar todas as etapas do estudo.

Participaram do estudo 50 indivíduos, funcionários da universidade e voluntários de maneira geral, com média de idade de 27,1 anos (idade mínima de 18 anos, máxima de 56 anos e desvio-padrão de 7,9 anos), 25 homens e 25 mulheres, sendo esta uma amostra de conveniência. A estimativa da amostra teve como base outros estudos que também investigaram os efeitos do exercício de oscilação oral de alta frequência sonorizada, utilizando o Shaker*®* ^(9,19)^.

Os participantes foram distribuídos, de acordo com a somatória obtida pela Escala de Sintomas Vocais (ESV)^(23)^, em quatro grupos: homens com sintomas vocais (ESV ≥ 16 pontos), homens sem sintomas vocais (ESV < 16 pontos), mulheres com sintomas vocais (ESV ≥ 16 pontos) e mulheres sem sintomas vocais (ESV < 16 pontos). A ESV é um protocolo de autoavaliação vocal e fornece informações sobre a funcionalidade, o impacto emocional e os sintomas físicos que um problema vocal pode gerar na vida do indivíduo^(23)^. A ESV consta de 30 perguntas sobre sintomas vocais, com cinco opções de resposta: nunca, raramente, às vezes, quase sempre e sempre. Cada questão é pontuada de 0 (nunca) a 4 (sempre). Indivíduos que atingiram pontuação igual ou maior que 16 foram incluídos no grupo “com sintomas vocais”^(23)^.

O grupo de homens com sintomas vocais foi composto por 13 indivíduos, com média de idade de 24,6 anos, desvio padrão (DP) de 3,8, mínimo de 19 e máximo de 34 anos e pontuação média na ESV de 26,46 (DP=10,33). O grupo de homens sem sintomas vocais foi constituído por 12 indivíduos, com média de idade de 25,1 anos, DP de 6,0, mínimo de 20 e máximo de 42 anos e pontuação média na ESV de 9,08 (DP=3,23). Com relação às mulheres, o grupo com sintomas vocais foi composto por 13 indivíduos, com média de idade de 29,5, DP de 9,5, mínimo de 19 e máximo de 53 anos e pontuação média na ESV de 29,00 (DP=13,33). O grupo de mulheres sem sintomas vocais constituiu-se de 12 indivíduos com média de idade de 29 anos, DP de 10,1, mínimo de 21 e máximo de 56 anos e pontuação média na ESV de 10,33 (DP=3,98).

### Procedimentos

Primeiramente, os participantes preencheram o protocolo ESV^(23)^. Em sequência foi realizada a gravação da voz do participante, em computador *notebook* com processador AMD Ryzen 5 3500U, placa de som Dolby áudio e microfone Lesson® HD 74 unidirecional (cardioide), devidamente conectado ao computador, posicionado lateralmente à boca do participante a uma distância de cinco centímetros, com taxa de amostragem de 44000 Hz e 16 bits por amostra. A coleta de dados foi realizada em sala acusticamente tratada com o participante sentado em postura ereta, tanto para a gravação da voz como para a realização do exercício.

A gravação da voz dos participantes constou da emissão sustentada da vogal /ɛ/ prolongada pelo tempo máximo de fonação e a contagem de 1 a 10. Foram considerados cinco segundos de execução da vogal /ɛ/, sendo que, para a análise acústica da voz, desprezaram-se os trechos iniciais e finais das amostras da vogal, que geralmente correspondem a um período de instabilidade natural da voz, mantendo-se o trecho médio, com duração entre três e quatro segundos.

Na sequência, após as emissões vocais (vogal sustentada e contagem), cada participante classificou, por meio da Escala Visual Numérica (EVN), o nível de desconforto vocal, indicando sua autopercepção vocal naquele momento. A EVN é composta por uma linha com numeração de 0 a 10, na qual em uma extremidade consta o número 0, que representa “nenhum desconforto” e, na outra extremidade, apresenta o número 10, que representa “máximo desconforto”. Foi explicado ao participante que “desconforto vocal” é a característica de desconforto, esforço ou cansaço que ele perceba sobre sua emissão no presente momento. O participante foi instruído a marcar o número que correspondia à autopercepção sobre o desconforto vocal naquele momento, para ser comparado posteriormente à prática do exercício.

Em seguida, os participantes realizaram um exercício que consistia em soprar o bocal do *Shaker®,* modelo *Classic*, emitindo a vogal /u/, na frequência habitual e com fluxo aéreo expiratório contínuo durante três minutos^(18)^, realizando pausas respiratórias quando necessárias. Os participantes realizaram as emissões em posição sentada em uma cadeira com costas e pés apoiados. O *Shaker®* foi mantido entre os lábios pelo próprio participante, segurando com uma das mãos, de maneira a manter um ângulo de 90º entre o dispositivo e o filtro labial^(9)^. Não foi realizado treinamento prévio, porém todos os participantes receberam orientação quanto à execução correta do exercício.

Após a prática por três minutos, uma nova gravação sequencial foi realizada da vogal /ɛ/ sustentada, contagem de 1 a 10 e classificação da autopercepção quanto ao desconforto vocal, por meio da EVN.

As gravações realizadas foram submetidas à análise acústica, por meio do *software* Voxmetria, versão 5,2. Os parâmetros acústicos analisados foram: média da frequência fundamental (f0) medida em Hz, ruído (dB), *glottal to noise excitation (GNE dB), jitter (%), shimmer (%),* proporção harmônico-ruído (dB) e as medidas cepstrais *Cepstral Peak Prominence* (CPP) e o *Cepstral Peak Prominence Smoothed* (CPPS). As medidas cepstrais foram obtidas por meio do *software* Praat, seguindo as recomendações propostas pela literatura^(24)^. Assim, foi realizada a análise comparativa de cada parâmetro acústico entre os períodos pré-exercício e pós-exercício.

As gravações também foram submetidas à análise perceptivo-auditiva realizada por três fonoaudiólogos, especialistas em voz e com experiência em análise perceptivo-auditiva da voz. Os áudios foram renomeados em “voz A e voz B” para deixar cega a análise e enviados em ordem aleatória para os fonoaudiólogos juízes, assim, tais fonoaudiólogos, ao analisarem os pares de vozes, não sabiam se cada voz analisada era pré ou pós-exercício. Todas as análises foram realizadas pelos juízes de forma independente e, durante a avaliação perceptivo-auditiva, os juízes foram orientados a escutar as amostras de voz quantas vezes considerassem necessário. Os fones de ouvido e computadores utilizados pelos juízes não foram do mesmo modelo ou marca.

A análise perceptivo-auditiva foi realizada por tarefa de comparação, portanto cada juiz deveria analisar, ao ouvir um par de vozes, se a segunda emissão (Voz B) "melhorou", "piorou" ou estava "sem alteração" em relação à primeira (voz A).

Quando houve discordância nas respostas dos três juízes para o mesmo par de vozes, um quarto avaliador, fonoaudiólogo especialista em voz, com mais de 20 anos de experiência na área, avaliou as vozes para a definição da resposta mais concordante. Esta análise foi realizada para sete pares de vozes.

A fim de se determinar a concordância intra-avaliadores, 20% da amostra foi replicada aleatoriamente. Foi utilizada a plataforma do Google Forms para organização e obtenção das respostas dos fonoaudiólogos juízes.

### Análise de dados

A análise estatística dos dados foi realizada por meio do programa estatístico MINITAB, versão 17. Primeiramente foi realizada análise descritiva dos dados com medidas de tendência central e de dispersão e, para a variável categórica (análise perceptivo-auditiva), o cálculo das frequências absolutas e relativas. Posteriormente, foi utilizado o teste de Anderson-Darling para verificar a normalidade da amostra. Para comparação das medidas acústicas de frequência fundamental (f0 Hz), ruído (dB); *glottal to noise excitation (GNE dB); jitter (%); shimmer (%);* proporção harmônico-ruído (dB); CPP e CPPs; e da autopercepção vocal nos momentos pré-execução do exercício e pós-execução do exercício para cada grupo, utilizou-se o teste paramétrico T-pareado ou não paramétrico de Wilcoxon. Para a comparação dos quatro grupos para a variável categórica da avaliação perceptivo-auditiva, foi utilizado o teste Qui-quadrado. Nesta análise a variável “piorou” foi retirada por sua baixa frequência de observações (quatro em cada tarefa – vogal sustentada e contagem). Foi utilizado o nível de confiança de 95% em todas as análises. Foi calculado o poder de teste para as variáveis que não apresentaram resultados estatisticamente significantes e o tamanho do efeito para aquelas com p<0,05 pelo programa G-Power. O tamanho do efeito foi considerado insignificante quando menor que 0,19; pequeno entre 0,20 e 0,49; médio para valores entre 0,50 e 0,79 e grande quando maior que 0,80^(25)^.

A concordância intra-avaliadores na avaliação perceptivo-auditiva foi avaliada por meio da estatística AC1 de Gwet, no *software* R versão 3.3.1. O grau de concordância foi analisado considerando-se: valores inferiores a zero - concordância ausente; 0 a 0,20 - concordância pequena; de 0,21 a 0,40 - concordância fraca; de 0,41 a 0,60 - concordância moderada; de 0,61 a 0,80 - concordância boa; e de 0,81 a 1,00 - concordância quase perfeita^(26)^.

## RESULTADOS

A Tabela 1 apresenta as medidas acústicas e a autopercepção do desconforto vocal do grupo de homens com sintomas vocais antes e após o exercício. Observou-se diferença estatisticamente significativa para *jitter* (p=0,048) e autopercepção do desconforto vocal (p=0,009), com redução dos valores dessas duas variáveis após o exercício.

**Tabela 1:** Medidas acústicas e autopercepção do desconforto vocal pré e pós-exercício em homens com sintomas vocais (n=13)

**Variável**	**Pré-exercício**							**Pós-exercício**							**Valor de p**	**1-β**	**Tamanho do efeito**
	**Média**	**DP**	**Mediana**	**1º quartil**	**3º quartil**	**Mínimo**	**Máximo**	**Média**	**DP**	**Mediana**	**1º quartil**	**3º quartil**	**Mínimo**	**Máximo**			
Frequência Fundamental (Hz)	108,62	16,57	113,22	99,77	118,20	70,72	130,08	117,75	12,43	116,29	110,07	126,09	96,64	134,68	0,232^(A)^	0,827	-
Ruído (dB)	1,68	0,79	1,61	1,17	1,79	0,53	3,54	1,38	0,65	1,34	1,00	1,84	0,45	2,52	0,106^(A)^	0,415	-
GNE (dB)	0,73	0,21	0,67	0,66	0,78	0,44	1,26	0,72	0,16	0,73	0,61	0,81	0,45	0,95	0,667^(A)^	0,672	-
PHR (dB)	7,89	4,83	8,52	6,21	10,82	1,04	16,31	8,54	5,66	9,91	2,45	11,27	0,52	17,6	0,711^(A)^	0,736	-
Jitter (%)	5,52	10,13	1,92	1,26	3,87	0,26	38,15	4,08	7,28	2,26	0,53	3,67	0,25	27,72	0,048*^(A)^	-	0,159
Shimmer (%)	33,0	26,19	23,75	19,41	36,49	12,64	114,10	20,75	9,15	23,25	11,37	24,55	6,73	36,23	0,507^(B)^	0,889	-
CPP vogal	23,18	3,38	23,56	21,35	24,97	17,35	28,13	24,83	3,11	24,94	23,08	27,52	19,56	29,49	0,055^(A)^	0,409	-
CPP contagem	19,64	1,20	19,41	19,15	20,55	17,41	21,71	19,78	1,16	19,99	18,79	20,49	17,90	21,82	0,734^(A)^	0,756	-
CPPS vogal	12,12	2,55	12,74	11,22	14,09	7,26	15,94	13,45	2,56	12,95	11,75	15,83	8,91	17,47	0,061^(A)^	0,447	-
CPPS contagem	10,49	1,41	10,69	10,18	11,27	6,6	12,21	10,39	1,29	10,42	9,66	11,52	8,15	11,94	0,713^(^A^)^	0,723	-
Autopercepção do desconforto vocal	2,38	2,14	2,0	0	4,0	0	6,0	0,61	0,77	0	0	1,0	0	2,0	0,009*(B^)^	-	0,943

Teste T pareado^(A)^;

Teste de Wilcoxon^(B)^

* Valor de p significante (p≤ 0,05)

**Legenda:** GNE=*Glottal to noise excitation*; PHR=Proporção harmônico-ruído; CPP=*Cepstral Peak Prominence;* CPPS=*Cepstral Peak Prominence-Smoothed*; 1-β=Poder de teste

A Tabela 2 apresenta as medidas acústicas e a autopercepção do desconforto vocal do grupo de homens sem sintomas vocais antes e após o exercício. Observou-se diferença estatisticamente significante para *shimmer* (p=0,042) e autopercepção do desconforto vocal (p=0,022), com redução dos valores para ambas as variáveis após o exercício.

**Tabela 2:** Medidas acústicas e autopercepção do desconforto vocal pré e pós-exercício em homens sem sintomas vocais (n=12)

**Variável**	**Pré-exercício**							**Pós-exercício**							**Valor de p**	**1-β**	**Tamanho do efeito**
	**Média**	**DP**	**Mediana**	**1º quartil**	**3º quartil**	**Mínimo**	**Máximo**	**Média**	**DP**	**Mediana**	**1º quartil**	**3º quartil**	**Mínimo**	**Máximo**			
Frequência Fundamental (Hz)	104,97	39,23	116,44	103,42	122,40	77,40	162,11	102,79	42,17	108,2	97,92	123,71	72,01	177,12	0,820^(A)^	0,823	-
Ruído (dB)	1,61	0,77	1,88	0,69	2,22	0,55	2,49	1,51	0,90	1,21	‘0,67	2,31	0,48	2,92	0,666^(B)^	0,690	-
GNE (dB)	0,64	0,17	0,59	0,50	0,72	0,46	0,92	0,69	0,22	0,76	0,50	0,89	0,35	0,94	0,388^(B)^	0,532	-
PHR (dB)	12,13	8,11	10,97	8,43	18,17	3,70	26,49	12,60	7,09	11,78	10,49	16,50	0,63	23,56	0,811^(A)^	0,815	-
Jitter (%)	3,19	3,80	1,33	0,70	4,3	0,34	9,84	3,02	4,16	1,00	0,59	3,43	0,2	14,38	0,290^(B)^	0,295	-
Shimmer (%)	24,58	13,79	20,98	15,71	35,97	3,22	47,27	17,91	10,33	16,33	11,32	20,68	5,05	39,53	0,042*^(A)^	-	0,537
CPP vogal	24,31	4,47	24,04	21,35	27,25	17,47	31,43	25,37	4,29	25,84	21,23	28,83	18,65	30,83	0,148^(A)^	0,271	-
CPP contagem	20,0	1,60	20,36	19,01	21,21	17,10	21,92	19,86	1,27	20,04	19,36	20,61	17,55	21,58	0,695^(A)^	0,710	-
CPPS vogal	13,11	3,59	12,68	10,60	15,66	7,01	18,81	14,08	3,57	14,43	10,53	17,40	8,55	18,59	0,078^(^A^)^	0,191	-
CPPS contagem	10,29	1,32	10,40	10,11	11.19	6,97	11,81	10,25	1,31	10,51	9,70	10,91	6,76	11,70	0,814^(B)^	0,815	-
Autopercepção do desconforto vocal	1,17	1,19	1,0	0	2,0	0	3,0	0,25	0,45	0	0	0,25	0	1	0,022*(B^)^	-	0,884

Teste T pareado^(A)^;

Teste de Wilcoxon^(B)^

* Valor de p significante (p ≤ 0,05)

**Legenda:** GNE=*Glottal to noise excitation*; PHR=Proporção harmônico-ruído; CPP=*Cepstral Peak Prominence;* CPPS=*Cepstral Peak Prominence-Smoothed*, 1-β=Poder de teste

As medidas acústicas e a autopercepção do desconforto vocal do grupo de mulheres com sintomas vocais, nos momentos antes e após o exercício, encontram-se na Tabela 3. Após o exercício, houve aumento da frequência fundamental (p=0,010) e da proporção harmônico-ruído (p=0,011), bem como do CPP (p=0,002) e CPPS da vogal sustentada (p=0,014); além de diminuição do *jitter* (p=0,014) e do desconforto vocal (p=0,002).

**Tabela 3:** Medidas acústicas e autopercepção do desconforto vocal pré e pós-exercício em mulheres com sintomas vocais (n=13)

**Variável**	**Pré-exercício**							**Pós-exercício**							**Valor de p**	**1-β**	**Tamanho do efeito**
	**Média**	**DP**	**Mediana**	**1º quartil**	**3º quartil**	**Mínimo**	**Máximo**	**Média**	**DP**	**Mediana**	**1º quartil**	**3º quartil**	**Mínimo**	**Máximo**			
Frequência Fundamental (Hz)	196,51	27,16	205,94	179,58	212,67	130,37	239,88	207,97	23,93	204,72	200,14	219,72	158,24	253,04	0,010*^(A)^	-	0,446
Ruído (dB)	1,28	0,65	1,06	0,82	1,78	0,39	2,59	1,09	0,57	1,09	0,56	1,30	0,41	2,08	0,080^(A)^	0,242	-
GNE (dB)	0,75	0,16	0,80	0,63	0,86	0,43	0,96	0,79	0,14	0,79	0,74	0,92	0,55	0,96	0,089^(A)^	0,216	-
PHR (dB)	9,67	7,54	7,47	5,21	13,45	0,66	28,19	12,67	7,01	11,78	6,37	15,02	5,54	27,93	0,011*^(A)^	-	0,411
Jitter (%)	3,59	4,24	2,11	0,73	5,12	0,19	14,12	1,80	2,73	1,51	0,19	2,06	0,08	10,36	0,014*^(B)^	-	0,480
Shimmer (%)	16,60	12,66	12,43	9,05	16,1	2,1	49,05	10,59	6,93	8,82	5,78	13,00	2,56	24,86	0,184^(B)^	0,700	-
CPP vogal	23,56	2,29	23,26	21,85	24,75	21,01	28,53	24,36	2,35	23,47	22,90	26,49	20,89	28,22	0,002*^(B)^	-	0,345
CPP contagem	19,19	1,45	19,45	18,23	19,88	16,71	21,94	19,40	0,85	19,41	19,13	19,95	17,79	20,79	0,616^(A)^	0,674	-
CPPS vogal	12,81	2,47	12,09	11,47	14,4	9,47	17,84	13,63	2,39	12,92	12,06	16,22	10,27	17,29	0,014*(B^)^	-	0,337
CPPS contagem	9,77	0,92	9,90	9,52	10,24	7,32	11,04	10,05	0,80	9,91	9,48	10,48	8,99	11,46	0,198^(^A^)^	0,443	-
Autopercepção do desconforto vocal	4,15	2,54	5,0	2,0	6,0	1,0	8,0	1,61	2,06	0	0	3,0	0	5,0	0,002*^(B)^	-	1,087

Teste T pareado^(A)^;

Teste de Wilcoxon^(B)^

* Valor de p significante (p≤0,05)

**Legenda:** GNE=*Glottal to noise excitation*; PHR=Proporção harmônico-ruído; CPP=*Cepstral Peak Prominence;* CPPS=*Cepstral Peak Prominence-Smoothed*, 1-β=Poder de teste

A Tabela 4 apresenta as medidas acústicas e a autopercepção do desconforto vocal do grupo de mulheres sem sintomas vocais antes e após a realização do exercício. Houve diferença significativa para as variáveis GNE (p=0,013) e autopercepção do desconforto vocal (p=0,009), sendo observados aumento do GNE e diminuição do desconforto vocal.

**Tabela 4:** Medidas acústicas e autopercepção do desconforto vocal pré e pós-exercício em mulheres sem sintomas vocais (n=12)

**Variável**	**Pré-exercício**							**Pós-exercício**							**Valor de p**	**1-β**	**Tamanho do efeito**
	**Média**	**DP**	**Mediana**	**1º quartil**	**3º quartil**	**Mínimo**	**Máximo**	**Média**	**DP**	**Mediana**	**1º quartil**	**3º quartil**	**Mínimo**	**Máximo**			
Frequência Fundamental (Hz)	237,24	89,10	212,44	189,59	229,32	184,08	273,47	214,47	22,18	209,72	201,67	232,54	181,09	253,19	0,733^(A)^	0,831	-
Ruído (dB)	1,30	0,71	1,30	0,75	1,65	0,42	2,87	1,02	0,40	1,03	0,67	1,25	0,49	1,67	0,085^(A)^	0,400	-
GNE (dB)	0,72	0,16	0,74	0,66	0,87	0,36	0,91	0,81	0,10	0,81	0,75	0,90	0,65	0,94	0,013*^(A)^	-	0,643
PHR (dB)	13,57	7,38	13,09	10,61	17,29	2,22	26,52	14,87	7,48	13,52	10,08	18,02	4,72	31,50	0,147^(A)^	0,212	-
Jitter (%)	1,94	2,54	1,02	0,39	1,65	0,13	7,40	1,91	2,46	0,70	0,54	1,98	0,14	7,13	0,845^(B)^	0,845	-
Shimmer (%)	10,76	4,81	11,10	7,45	14,37	2,54	16,97	12,47	6,50	12,15	9,28	15,68	2,35	25,68	0,335^(A)^	0,536	-
CPP vogal	23,04	2,12	23,55	21,09	24,75	19,74	25,59	23,97	1,97	23,80	22,60	25,36	20,60	27,68	0,060^(A)^	0,332	-
CPP contagem	18,86	2,12	18,83	17,36	20,63	15,90	21,54	18,49	1,75	18,46	17,29	20,11	15,83	20,65	0,181^(A)^	0,263	-
CPPS vogal	12,71	2,07	12,82	10,79	14,26	10,04	15,62	13,48	2,06	13,73	11,97	14,58	10,13	17,47	0,066^(^A^)^	0,260	-
CPPS contagem	9,31	1,67	10,09	8,33	10,25	6,33	11,08	9,18	1,80	9,98	7,86	10,70	6,06	10,87	0,367^(B)^	0,382	-
Autopercepção do desconforto vocal	2,50	1,57	2,50	2,0	3,0	0	5,0	0,83	1,40	0,5	0	1,0	0	5,0	0,009*(B^)^	-	1,119

Teste T pareado^(A)^;

Teste de Wilcoxon^(B)^

* Valor de p significante (p≤0,05)

Legenda: GNE=*Glottal to noise excitation*; PHR=Proporção harmônico-ruído; CPP=*Cepstral Peak Prominence;* CPPS=*Cepstral Peak Prominence-Smoothed*, 1-β=Poder de teste

A Tabela 5 apresenta o resultado da análise perceptivo-auditiva para os quatro grupos estudados, considerando a voz no momento pós-exercício em comparação com o momento pré-exercício. Obteve-se 100% de concordância intra-avaliador para os três juízes. Dessa forma, as respostas dos três avaliadores foram consideradas para análise perceptivo-auditiva da voz, sendo utilizado o valor da moda das respostas dadas pelos fonoaudiólogos. Para a maior parte dos participantes, não foi verificada alteração vocal na avaliação perceptivo-auditiva, sem diferença entre os grupos (p=0,379) para a vogal sustentada, porém com diferença entre os grupos para a fala encadeada (p=0,003). O grupo de mulheres com sintomas vocais foi o grupo que apresentou menos modificações vocais após a realização do exercício de oscilação oral de alta frequência sonorizada.

**Tabela 5:** Resultado da comparação da voz no momento pós-exercício com o momento pré-exercício, por meio da análise perceptivo-auditiva

**Resultado**	**Vogal Sustentada**		**Contagem**	
	**N**	**%**	**N**	**%**
Homens com sintomas vocais (n=13)				
Melhorou	9	69,23	6	46,15
Sem alteração	4	30,77	7	53,85
Piorou	0	0	0	0
Homens sem sintomas vocais (n=12)				
Melhorou	5	41,67	3	25
Sem alteração	6	50	9	75
Piorou	1	8,33	0	0
Mulheres com sintomas vocais (n=13)				
Melhorou	5	38,46	0	0
Sem alteração	7	53,85	13	100
Piorou	1	7,69	0	0
Mulheres sem sintomas vocais (n=12)				
Melhorou	3	25	0	0
Sem alteração	7	58,33	8	66,67
Piorou	2	16,67	4	33,33

**Legenda:** N=frequência absoluta; %=frequência relativa

## DISCUSSÃO

A presente pesquisa aponta que a OOAFS realizada com o Shaker*®* modelo *Classic* promoveu mudanças vocais positivas observadas na análise acústica e de autopercepção em adultos, sendo que tais mudanças variaram conforme sexo e presença de sintomas vocais. Mulheres com sintomas vocais apresentaram resultados positivos em um maior número de medidas acústicas após o exercício, e todos os grupos autorreferiram menor desconforto vocal, sugerindo que a OOAFS realizada com o Shaker*®* modelo *Classic* é um exercício seguro.

No grupo de homens com sintomas vocais, houve redução do *jitter* e diminuição do desconforto vocal após o exercício de oscilação oral de alta frequência sonorizada. Considerando que *jitter* é uma variação da frequência fundamental a curto prazo e determina a constância do sistema fonatório, valores menores referentes a essa medida acústica após o exercício sugerem uma maior estabilidade e menor perturbação da frequência fundamental^(27)^. Saters et al.^(19)^, utilizando o New Shaker®, apontaram diminuição dos sintomas vocais e laríngeos em homens com sintomas vocais, porém, sem diferença dos parâmetros acústicos, após a realização de três minutos do exercício. Já Marotti et al.^(28)^, também utilizando o New Shaker®, não encontraram mudanças perceptivo-auditivas significativas nessa população, tanto na análise da vogal como na contagem de números, mas verificaram sensações imediatas positivas autorreferidas relacionadas a voz, laringe, respiração e articulação. Sendo assim, os estudos concordam quanto aos efeitos positivos da OOAFS enquanto ETVSO na redução do desconforto vocal. Tais resultados sugerem que esse exercício favorece o equilíbrio entre as contrações musculares da laringe e o fluxo aéreo expirado^(7)^, aliviando sintomas de desconforto vocal.

No grupo de homens sem sintomas vocais, houve diminuição de *shimmer*, além de redução do desconforto vocal autorrelatado. O *shimmer* classifica a variabilidade da amplitude da onda sonora a curto prazo^(27)^, sendo, portanto, uma medida da perturbação da intensidade da voz. A execução do exercício pesquisado é caracterizada pela oclusão parcial na região anterior do trato vocal. É lícito supor que o Shaker® aumenta a interação fonte-filtro, provoca a diminuição da pressão glótica e, consequentemente, favorece a periodicidade de amplitude de vibração das pregas vocais, além de melhorar o conforto vocal^(9,18,19)^. Um estudo^(21)^, utilizando o New Shaker®, encontrou diminuição do *shimmer* em mulheres sem sintomas laríngeos após cinco minutos de execução do exercício de OOAFS, o que pode indicar que, em indivíduos sem sintomas vocais, o exercício promove maior estabilidade da vibração das pregas vocais. Os achados da presente pesquisa, em relação à melhora do desconforto vocal em homens sem sintomas vocais, concordam com os achados de outra pesquisa^(20)^ que, utilizando o *New* Shaker®, verificou, também em homens sem sintomas vocais, diminuição dos sintomas laríngeos após a execução deste mesmo exercício.

O grupo de mulheres com sintomas vocais foi o que apresentou mudanças acústicas positivas em um maior número de variáveis, tendo sido observado aumento da frequência fundamental, da proporção harmônico-ruído, das medidas cepstrais extraídas da vogal, além de diminuição de *jitter* e do desconforto vocal. Um estudo, utilizando o Shaker® *Classic*, com mulheres com e sem sintomas vocais, encontraram apenas diminuição do *jitter* e melhora do desconforto vocal^(18)^. Outro estudo, que empregou o *New* Shaker®, com mulheres normofônicas e disfônicas verificaram melhora de sintomas vocais e laríngeos^(19)^. O aumento da frequência fundamental foi verificado, para indivíduos sem queixa vocal, no estudo de Saters et al.^(19)^, utilizando o *New* Shaker®, como resposta ao exercício de OOAFS, fato também apontado por estudos com outros exercícios de trato vocal semiocluído^(8)^.

Verificou-se aumento dos valores de CPP e CPPs avaliados pela vogal, no grupo de mulheres com sintomas vocais. O CPP é uma medida da análise cepstral, procedimento de extração da frequência fundamental do espectro de uma onda sonora^(29)^. O cepstro mostra, sob forma de gráfico, o quanto os harmônicos espectrais, em particular a frequência fundamental vocal, são individualizados e se destacam do ruído de fundo^(29)^. Quanto mais regulares os picos harmônicos e quanto maior a periodicidade e a energia geral do sinal de voz, maior a amplitude do pico cepstral^(29)^. Dessa forma, o aumento de CPP e CPPs, verificado em mulheres com sintomas vocais, após o exercício, é um indicativo da melhora da qualidade vocal, decorrente do aumento da estrutura harmônica da voz. Estudos apontam que as medidas cepstrais vêm demonstrando potencial especialmente para avaliação de vozes com ampla faixa de desvio^(30,31)^, o que justifica o fato de apenas o grupo de mulheres com sintomas vocais ter apresentado aumento dessa medida. Vale ressaltar que os valores de CPP e CPPs da vogal sustentada no grupo de homens com sintomas vocais ficaram próximos do ponto de corte para significância estatística. Pode ser que um tempo diferente de execução do exercício para os homens com sintomas vocais traria resultados positivos para as medidas cepstrais. Antonetti et al.^(17)^ verificaram que tanto homens quanto mulheres sem alterações vocais tiveram aumento de CPPs a partir de um minuto do exercício de OOAFS com o *New* Shaker®, com manutenção após três minutos, mas não foram encontrados estudos que avaliassem medidas cepstrais em homens com sintomas ou mesmo disfônicos antes e após o exercício.

Para o grupo de mulheres sem sintomas vocais, observou-se aumento do GNE e diminuição do desconforto vocal. Enquanto a literatura concorda que o exercício de OOAFS para mulheres normofônicas, com o dispositivo *New* Shaker®, promove melhora de sintomas vocais^(9,19)^ e promove conforto vocal^(18)^, a variação de GNE não foi um achado verificado em nenhum outro estudo com o Shaker®. A medida acústica GNE está diretamente relacionada à presença de rugosidade e soprosidade^(32)^. Valores menores de GNE podem indicar fechamento glótico ineficaz, com presença de ruído na voz e possível perda da intensidade^(32)^. Nesse sentido, o aumento do GNE é um resultado positivo para este grupo.

Na análise perceptivo-auditiva, para a maior parte dos participantes, não foi verificada modificação vocal após a realização do exercício. Apesar de o grupo de homens com sintomas vocais ter apresentado maior número de indivíduos com melhora vocal no julgamento dos juízes, não houve diferença significativa entre os grupos. O resultado da presente pesquisa concorda com a literatura. Um estudo^(9)^ com mulheres idosas apontou maior quantidade de indivíduos com manutenção da qualidade vocal após a execução do exercício por três minutos. Outros estudos com adultos sem queixa vocal^(16)^ e com adultos disfônicos^(22)^ também verificaram ausência de mudanças na análise perceptivo-auditiva da voz após realização do exercício, por três^(16,22)^, cinco^(22)^ e sete minutos^(22)^. Os três estudos^(9,16,22)^ foram realizados com o *New* Shaker®.

Ressalta-se que o tempo ideal de execução do exercício pode não ser o mesmo para ambos os sexos e isso pode ter influenciado os achados. A presente pesquisa pautou-se no estudo de Siqueira e colaboradores^(18)^ que avaliaram o tempo ideal de execução do exercício, para o Shaker *Classic*®, apenas para mulheres. Um outro estudo, com o *New* Shaker®, verificou diminuição significativa no parâmetro acústico de ruído vocal, considerando indivíduos saudáveis, após sete minutos de execução do exercício de OOAFS para homens^(21)^; ao passo em que, para as mulheres, a partir do terceiro minuto já se havia notado diferença significativa nos sintomas laringofaríngeos^(21)^. São necessários mais estudos, com o modelo *Classic*, que avaliem o efeito do exercício de OOAFS na população masculina e com maior tempo de execução, visando a melhor compreensão da dose ideal deste exercício vocal nessa população.

O tamanho de efeito variou de insignificante a grande, sendo que a autopercepção de desconforto vocal apresentou tamanho de efeito grande nos quatro grupos testados^(25)^. A literatura não apresenta o tamanho de efeito que pode ser considerado relevante para as variáveis pesquisadas. Nesse sentido, é importante que a Fonoaudiologia comece a se atentar para questões relacionadas à análise do tamanho de efeito, para um maior entendimento do tamanho de efeito clinicamente relevante para a área.

A literatura tem mostrado que exercícios de trato vocal semiocluído podem ser usados para treinamento voltados à melhoria da qualidade da voz e para aquecimento vocal^(9,19)^. Com isso, estudos já publicados e os resultados do presente estudo sugerem que este é um exercício seguro, sob os aspectos clínicos avaliados, pois não trouxe piora de desconforto vocal ou piora nos parâmetros controlados. Sugere-se, também, que futuras pesquisas avaliem o efeito do exercício de OOAFS em profissionais da voz.

Constituíram-se limitações do estudo o recrutamento por conveniência, o tamanho da amostra, que ao ser estratificada resultou em poucos indivíduos por grupo, por isso o poder do teste, para algumas variáveis, ficou abaixo do preconizado, bem como a ampla faixa etária da amostra, uma vez que mudanças vocais podem ocorrer no climatério^(33)^. Outra limitação importante refere-se ao fato de que a divisão dos grupos se baseou em uma escala de autoavaliação. Dessa forma, é possível que, dentre os indivíduos sem sintomas vocais, possam existir indivíduos disfônicos. Não constituiu variável da pesquisa a imagem laríngea, portanto, pesquisas futuras com o exame laringológico dos participantes podem trazer contribuições importantes sobre o efeito do exercício de oscilação oral de alta frequência sonorizada na função glótica. Sugerem-se, também, a pesquisa de medidas que possam fornecer informações sobre a relação fonte-filtro, como a relação alfa, L1-L0, por exemplo. Apesar das limitações, o estudo traz contribuições para a prática clínica fonoaudiológica, dado o rigor metodológico com que foi realizado e considerando-se a escassez de estudos que utilizaram o exercício com o Shaker *Classic*® e que incluíram a análise de medidas cepstrais. Sugere-se, ainda, a condução de estudos com amostras maiores, para que se verifique o real efeito do uso do exercício de OOAFS em indivíduos com diferentes condições clínicas e que apresentem maior validade externa.

Recomendam-se novas pesquisas que possam analisar o efeito do exercício de OOAFS em participantes com disfonia, bem como os efeitos do dispositivo a longo prazo. A associação de exames clínicos laringológicos a esses estudos também poderá enriquecer os achados, propiciando análise multidimensional da voz. Além disso, é importante que existam novas pesquisas comparando tempos diferentes de execução do exercício para homens e mulheres, bem como para diferentes condições clínicas e faixas etárias.

## CONCLUSÃO

O exercício de oscilação oral de alta frequência sonorizada, utilizando o dispositivo Shaker® modelo *Classic,* realizado por três minutos, foi capaz de promover mudanças acústicas imediatas positivas em todos os grupos analisados. Mudanças perceptivas não foram verificadas. Todos os grupos relataram redução do desconforto vocal.
